# A rare simultaneous coexistence of pancreatic gastrointestinal stromal tumor and esophageal schwannoma: a case report and review of literature

**DOI:** 10.3389/fonc.2024.1428910

**Published:** 2024-09-23

**Authors:** Xiaonan Yin, Hongxin Yang, Bo Zhang, Yuan Yin

**Affiliations:** ^1^ Gastric Cancer Center, Department of General Surgery, West China Hospital, Sichuan University, Chengdu, Sichuan, China; ^2^ Department of Gastrointestinal Surgery, The Affiliated Hospital of Guizhou Medical University, Guiyang, Guizhou, China

**Keywords:** gastrointestinal stromal tumor, schwannoma, case report, treatment, differential diagnosis

## Abstract

The concurrent presence of gastrointestinal stromal tumor and schwannoma is extremely rare, and its pathological characteristics remain unclear. This case report reported the diagnostic and treatment process of a patient with a pancreatic GIST coexisting with esophageal schwannoma, who was admitted to West China Hospital (Sichuan, China) in April 2015. The patient did not undergo surgical resection of the tumor but instead received an 8-year regimen of imatinib therapy, during which no tumor progression was observed. However, the patient developed pleural effusion as a result of the localized enlargement of the esophageal schwannoma, which exerted pressure on the right inferior pulmonary vein. This case report provides valuable clinical insights into this distinctive disease presentation.

## Background

Gastrointestinal stromal tumors (GISTs) arise from the mesodermal interstitial cells of Cajal (ICC), which are characterized by the expression of the c-KIT protein (1). They are the predominant mesenchymal neoplasms in the gastrointestinal tract, comprising approximately 80% of such instances (2). The stomach is the predominant site of occurrence (50-60%), followed by the small intestine, colorectum, and esophagus. Rare extra-gastrointestinal GISTs (EGISTs) are reported originating from omentum, mesentery and retroperitoneum (3). Primary pancreatic GISTs are exceptionally uncommon, accounting for approximately 5% of all EGISTs cases (4). Typically, the preoperative diagnosis of GISTs relies on abdominal and pelvic computed tomography (CT) scans or magnetic resonance imaging (MRI). Typical differential diagnoses for GISTs encompass leiomyoma, leiomyosarcoma, schwannoma, as well as certain uncommon mesenchymal tumors such as desmoid tumors, solitary fibrous tumors, inflammatory myfibroblastic tumors, perivascular epithelioid cell tumors, and inflammatory fibroid polyps (5). Endoscopic ultrasonography (EUS) is the optimal modality for characterizing subepithelial lesions, enabling detailed assessment of parameters like size, location, originating layer, echogenicity, and shape (6). Additionally, EUS-guided fine-needle biopsy (EUS-FNB) stands out as a highly effective technique for diagnosing gastrointestinal subepithelial lesions (7). Definitive diagnosis of these tumors usually requires postoperative pathological analysis and immunohistochemistry. In addition, molecular testing plays a crucial role in confirming the diagnosis of GISTs. In this study, we present a highly unusual case of pancreatic GIST coexisting with esophageal schwannoma, along with a review of relevant literature.

## Case report

**Figure 1:**
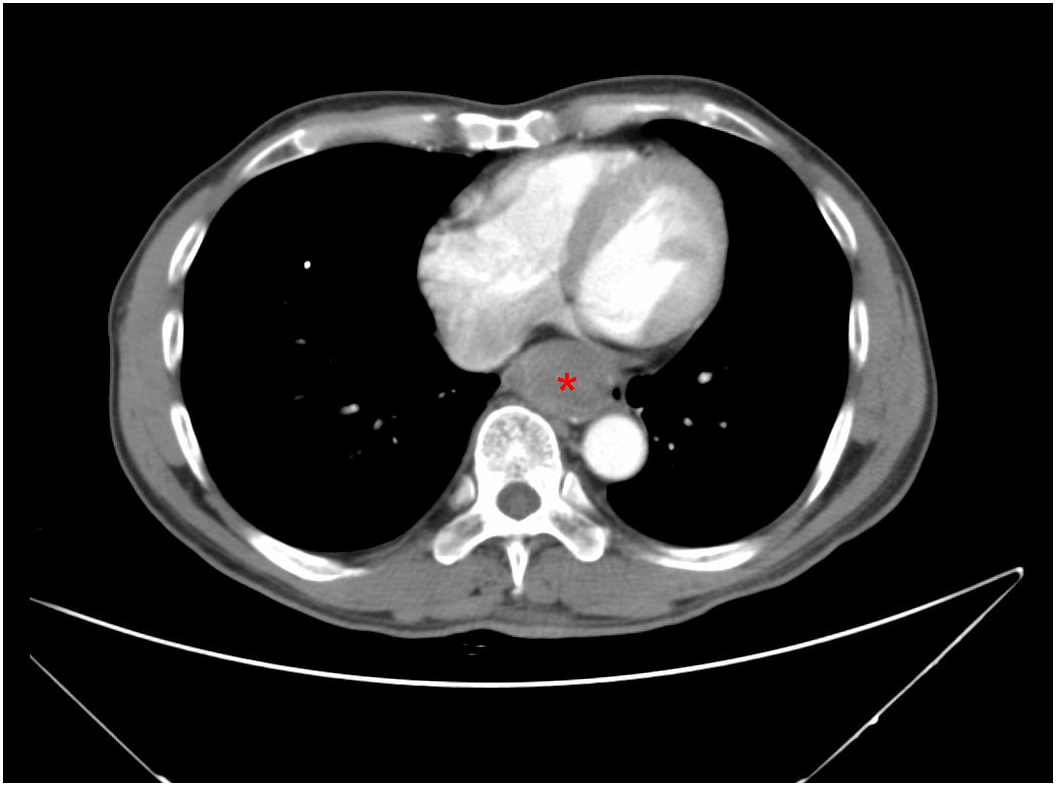
A CT scan of the chest reveals a 3.8-cm mass in the lower esophagus, exhibiting slight enhancement on the contrast-enhanced scan (red asterisk).

**Figure 2:**
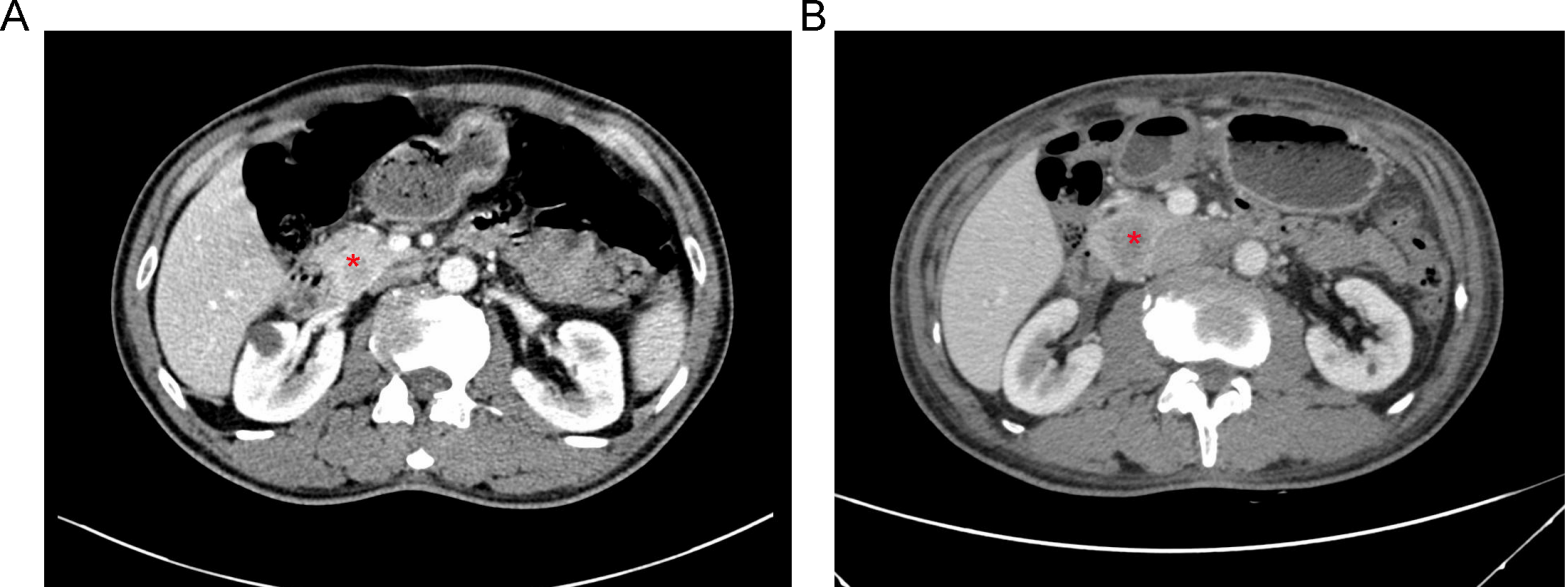
**(A)** An abdominal CT scan revealing a 3.4-cm mass with heterogeneous enhancement in the region of the pancreatic head (red asterisk). **(B)** Follow-up CT scan imaging six months after starting therapy with imatinib, showing no significant changes in tumor dimension or density (red asterisk).

**Figure 3:**
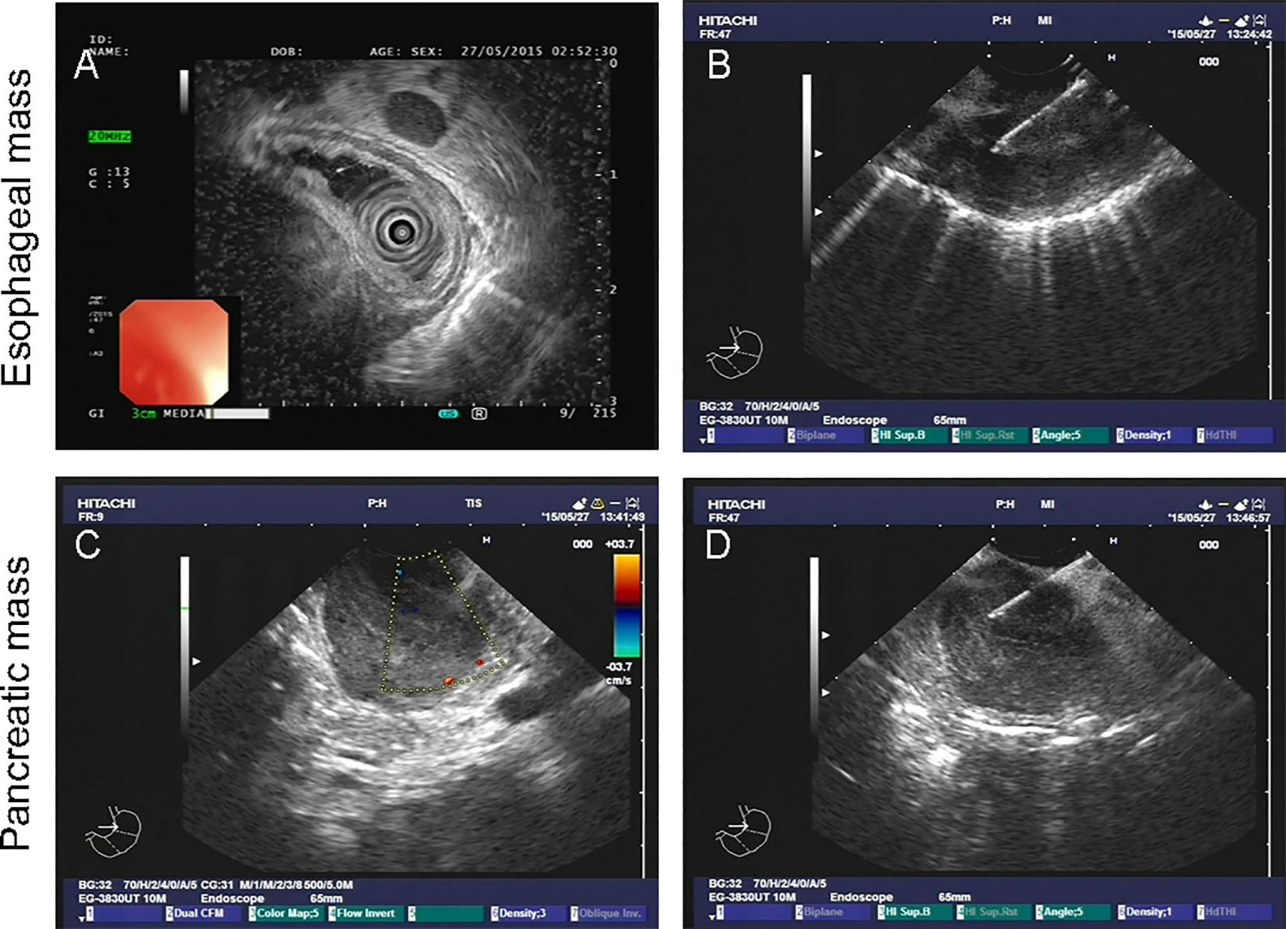
Endoscopic ultrasound-guided fine-needle biopsy of esophageal **(A, B)** and pancreatic mass **(C, D)**.

**Figure 4:**
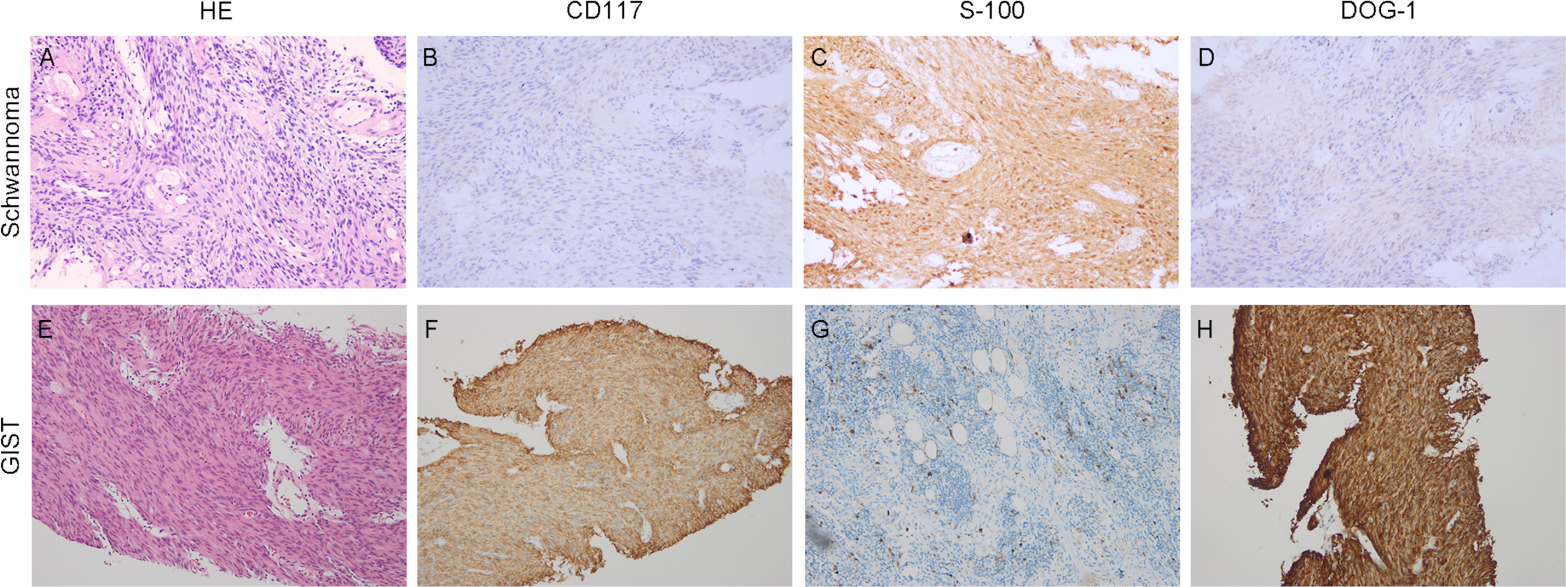
Histopathological findings. Hematoxylin-eosin (HE) and immunohistochemical staining. **(A–D)** (×20 objective) depict Schwannoma: **(A)** HE, **(B)** CD117 negative, **(C)** S100 positive, **(D)** DOG1 negative. **(E–G)** (×20 objective) illustrate GISTs: **(E)** HE, **(F)** CD117 positive, **(G)** S100 negative, **(H)** DOG1 positive.

A 61-year-old male patient presented at West China Hospital, Sichuan University in April 2015 with a chief complaint of “difficulty swallowing for six months.” He had no significant medical history or family history of cancer. Tumor markers, including carcinoembryonic antigen (CEA), carbohydrate antigen 19-9 (CA19-9), CA724, and CA153, were within normal ranges. A chest CT scan (**Figure 1**) revealed localized thickening of the lower esophagus, with a soft-tissue mass measuring approximately 38 x 30 x 40 mm within the lumen. The mass exhibited slight enhancement on the contrast-enhanced scan, leading to significant luminal narrowing in the corresponding esophageal segment. An abdominal CT scan (**Figure 2A**) showed a slightly low-density mass measuring 29 x 34 mm in the pancreatic head region. The enhancement pattern of the mass was uneven, with an unclear demarcation between the lesion and the pancreatic head parenchyma. An EUS with fine-needle aspiration (**Figures 3A, B**) using a standard 22G needle of the esophageal mass revealed a spindle cell neoplasm (**Figure 4A**). This neoplasm tested positive for the S-100 protein antibody (**Figure 4C**) and exhibited negative staining for CD117 (**Figure 4B**), DOG-1 (**Figure 4D**), CD34, smooth muscle actin, and desmin (data not shown). These results confirmed the diagnosis of a benign esophageal schwannoma. Subsequent EUS -guided fine-needle aspiration (**Figures 3C, D**) using a standard 22G needle of the pancreatic mass indicated a spindle cell neoplasm (**Figure 4E**). This tumor exhibited diffuse positivity for CD117 (**Figure 4F**) and DOG1 (**Figure 4H**), while testing negative for S-100 (**Figure 4G**), consistent with a diagnosis of GIST. Following the confirmed diagnosis of GIST, the patient started treatment with imatinib. A follow-up abdominal CT scan six months after commencing imatinib therapy showed no significant changes in tumor size or density within the pancreatic head region (**Figure 2B**). A pancreaticoduodenectomy (Whipple procedure) was recommended for the patient but was declined. Instead, the patient opted to persist with imatinib treatment and undergo regular CT scans every six months to monitor tumor progression. From October 2015 to December 2022, there were no significant changes observed in the esophageal schwannoma and pancreatic GIST based on CT scan findings. However, in May 2023, the patient was admitted to the hospital due to chest symptoms including tightness, fatigue, and anorexia. Chest CT scans (data not shown) revealed localized thickening of the lower esophagus and a soft-tissue mass within the lumen, measuring approximately 56 x 38 x 45 mm. The soft-tissue component exhibited uneven enhancement, causing significant narrowing of the esophageal segment, leading to right pleural effusion due to compression of the right inferior pulmonary vein. No significant changes were observed in the pancreatic GISTs based on the abdominal CT scan. Drainage of the right pleural effusion resulted in the extraction of 650 ml of light-yellow fluid. Following this intervention, the patient’s condition improved. The patient discontinued imatinib treatment and was advised to undergo chest and abdominal CT scans every six months. As of April 2024, there have been no indications of tumor progression in the patient.

## Discussion

To the best of our knowledge, the present case report describes the first case of pancreatic GIST coexisting with esophageal schwannoma. Both GISTs and gastrointestinal schwannomas are frequently located in the submucosal layer of the digestive tract, with the stomach being the most common site for both types of tumors (2, 8). Differential diagnosis via preoperative imaging poses challenges, necessitating postoperative pathological examination for accurate identification. While a previous study has documented the concomitant presence of GISTs and gastric schwannoma, the underlying mechanisms behind this simultaneous occurrence remain elusive (9). Various hypotheses have been proposed to elucidate the coexistence of GIST and schwannoma. Firstly, genetic mutations or combined genetic dysregulation may contribute to the development of synchronous gastric tumors. Secondly, shared initiating factors among distinct mesenchymal tumors could underlie the pathogenesis of both gastric GISTs and schwannoma. In our case, we believe that shared initiating factors within the mesenchyme are implicated in the pathogenesis of both GIST and schwannoma, given their common mesenchymal origins.

In our case report, the occurrence of both pancreatic GIST and esophageal schwannoma is exceptionally rare. Pancreatic tumors predominantly arise from epithelial tissue, with mesenchymal origins being less common, and spindle cell neoplasms in the pancreas are particularly uncommon. Primary pancreatic GIST is a rare entity, with only 57 cases reported in the literature prior to February 2023 (10). These tumors are typically located in proximity to the main pancreatic duct, major blood vessels, and the exocrine pancreas (11, 12). Pancreatic GISTs most commonly manifest in the pancreatic head, with abdominal pain being the primary presenting symptom (13). CT scans of pancreatic GISTs typically reveal well-defined solid masses with frequent cystic changes and associated hemorrhage (14–16). The diagnosis of pancreatic GISTs often relies on EUS-guided fine needle biopsy, with positive immunostaining for CD117 and DOG-1 confirming the diagnosis. Additionally, genetic mutation analysis is pivotal for diagnosing GISTs, as the majority harbor mutations in c-KIT and/or PDGFRA genes (17). Schwannomas are neuroectodermal tumors that arise from Schwann cells, which are the cells responsible for the myelin sheath surrounding peripheral nerves (18, 19). While gastrointestinal schwannomas primarily localize to the stomach, they can also occur, albeit rarely, in the colorectum, small intestine, esophagus, and gallbladder. Most schwannomas are benign and exhibit slow growth (20). Symptoms of esophageal schwannomas typically correlate with tumor size and location, often presenting as dysphagia and symptoms of esophageal reflux, with respiratory symptoms occurring when tumors attain significant size. Preoperative diagnosis of esophageal schwannomas is challenging, typically necessitating postoperative pathological examination. Immunohistochemical staining revealing positivity for S-100, a characteristic marker of Schwann cells, aids in confirming the diagnosis. Both GIST and schwannoma exhibit resistance to chemotherapy and/or radiotherapy. The standard treatment involves complete surgical resection with negative margins. Additionally, targeted therapy using tyrosine kinase inhibitors is essential for patients with intermediate and high-risk GISTs, as determined by postoperative risk classification (21). However, guidelines for risk assessment in predicting the prognosis of primary pancreatic GISTs are lacking due to their rarity. A recent study (10) conducted a comprehensive review of the available literature, identifying 57 cases of primary pancreatic GISTs. The study findings revealed that factors influencing the disease-free survival of pancreatic GISTs included histologic type, mitotic index, NIH risk category, and the use of adjuvant therapy. Another study proposed that pancreatic GISTs exhibit notable disparities in clinicopathological features when contrasted with gastric GISTs, demonstrating a more aggressive nature than their gastric counterparts (13). However, the sample size of pancreatic GISTs was relatively small, necessitating larger case series with extended follow-up data to gain deeper insights into the disease biology and outcomes. Furthermore, it is essential to acknowledge specific limitations of our study. The definitive surgical pathology remains unavailable in our case; consequently, the pancreatic origin of the gastrointestinal stromal tumor (GIST) cannot be confirmed with certainty.

## Conclusion

In conclusion, we present the first known case report detailing the concurrent presence of esophageal schwannoma and pancreatic GISTs, utilizing immunohistochemistry and histology at our institution. Diagnosing these rare and unique coexisting tumors can pose challenges, but immunochemical and mutation analyses prove valuable. Understanding the morphological and biological attributes of this coexistent condition was crucial for accurate diagnosis and appropriate treatment planning for future patients.

## Data Availability

The datasets used and/or analyzed during the current study are available from the corresponding author upon reasonable request. Requests to access the datasets should be directed to yinyuan10@wchscu.cn.
